# Rosmarinic Acid Ameliorates PM_2.5_-Induced Alterations in Gut Microbiota and Intestinal Inflammation in Broilers

**DOI:** 10.3390/ani16101428

**Published:** 2026-05-07

**Authors:** Ying Zhou, Bin Xu, Wen Deng, Linyi Wang, Shaoyu Li

**Affiliations:** Institute of Animal Husbandry and Veterinary Science, Henan Academy of Agricultural Sciences, Zhengzhou 450002, China; zhouying@hnagri.org.cn (Y.Z.); xubin@hnagri.org.cn (B.X.); dengwen@hnagri.org.cn (W.D.); wanglinyi@hnagri.org.cn (L.W.)

**Keywords:** broiler, gut microbiota, PM_2.5_, rosmarinic acid, TLR4/NF-κB

## Abstract

(1) Airborne fine particulate matter (PM_2.5_) poses a growing threat to poultry production by impairing intestinal health, disturbing microbial balance, and reducing growth performance. Rosmarinic acid (RA), a natural polyphenol with antioxidant, anti-inflammatory, and gut microbiota-regulating properties, can effectively maintain intestinal homeostasis. To date, its protective effects against PM_2.5_-induced intestinal injury in broilers remain largely unclear. This study investigated whether dietary RA supplementation mitigates intestinal damage and microbiota dysbiosis caused by PM_2.5_ in broilers and explored the related mechanisms. (2) A total of 144 21-day-old broilers were randomly allocated to three groups, control (CON), PM_2.5_ exposure (PM), and PM_2.5_ exposure plus rosmarinic acid (RA), with six replicates of eight broilers each. (3) Results indicated that PM_2.5_ exposure severely impaired growth performance, whereas dietary RA significantly increased average daily feed intake and average daily gain, decreased the feed-to-gain ratio, and elevated final body weight in broilers. RA significantly attenuated PM_2.5_-induced intestinal inflammation, as evidenced by reduced expression of inflammatory cytokines (IL-6 and IFN-γ) and downregulation of key components in the TLR4 signaling pathway (TLR4, MyD88, and NF-κB). Inhaled PM_2.5_ exposure impaired the intestinal epithelial barrier, marked by decreased mRNA levels of MUC2 and CLDN1 and increased caspase3 expression. Dietary RA treatment effectively restored these indicators, suggesting its role in maintaining epithelial integrity. Furthermore, RA reshaped the gut microbiota structure, altering both α- and β-diversity. Notably, RA led to a higher proportion of potentially health-promoting bacterial taxa, including *Lactobacillus*, *V9D2013_group*, and *Oscillospirales*, while reducing opportunistic pathogens like *Shuttleworthia*. (4) In conclusion, RA alleviates PM_2.5_-induced intestinal inflammation, reinforces the epithelial barrier, and modulates the intestinal microbiota in broilers, likely through inhibition of the TLR4/NF-κB signaling. These findings reveal a novel mechanism by which RA mitigates pollutant-induced intestinal injury via gut microbiota modulation and TLR4/NF-κB suppression, offering new insights into the gut–lung axis in avian species.

## Simple Summary

Exposure to airborne fine particulate matter (PM_2.5_) is a growing health concern not only for respiratory health but also for intestinal function. In this study, we investigated whether dietary supplementation with rosmarinic acid, a natural plant compound with known bioactive properties, could protect broilers from intestinal injury caused by inhaled PM_2.5_. Our results showed that PM_2.5_ exposure triggered intestinal inflammation and weakened the gut barrier in broilers. However, adding rosmarinic acid to their diet effectively reduced this inflammation, helped maintain intestinal integrity lining, and favorably modulated the gut microbiota composition by increasing beneficial bacteria like *Lactobacillus*. These findings suggest that rosmarinic acid has the potential to serve as a valuable dietary strategy to counteract the harmful effects of air pollution on intestinal health in poultry, offering new insights into the protective mechanisms involving the gut–lung axis.

## 1. Introduction

Epidemiological evidence links ambient air pollution to increased disease risk in humans and animals, with fine particulate matter (PM_2.5_)—a major airborne pollutant—threatening both environmental quality and public health, as well as livestock production [1]. Intensive poultry operations contribute ~50% of agricultural PM emissions [2], and commercial broiler houses (especially litter-based systems) have higher PM levels than swine or cattle barns. Even modern environmentally controlled facilities frequently exceed PM safety standards, with concentrations rising with broiler age and varying by season [3]. Beyond well-documented respiratory harm [4], it also adversely affects gastrointestinal health, correlating with disorders like inflammatory bowel disease and intestinal infections [5,6]. For broilers, elevated PM impairs immunity, reduces growth, and increases mortality, directly threatening production efficiency despite advanced housing technology.

Gut microbiota serves as a critical homeostatic regulator for maintaining host health. By preserving intestinal barrier integrity, modulating immune responses, and suppressing pathogen colonization, it establishes a complex and dynamic interactive network with the host, thereby promoting intestinal homeostasis and overall physiological balance [7,8]. Disruptions to the gut microbial ecosystem have been causally linked to disease development triggered by inhaled environmental pollutants [9,10]. Through mucociliary clearance, PM_2.5_ particles enter the intestine, where they disrupt gut microbiota by selectively enriching or depleting specific microbial taxa [11,12]. Evidence suggests that exposure to PM_2.5_ impairs intestinal barrier function [13,14], induces oxidative stress [15], and activates inflammatory pathways, notably the TLR4/NF-κB signaling cascade, orchestrating the secretion of key pro-inflammatory cytokines, namely Interleukin-6 (IL-6), Tumor Necrosis Factor-α (TNF-α), and Interleukin-1β (IL-1β) [14]. These cascading effects further disrupt the structure and function of the gut microbial ecosystem [11,12,16]. Given the established role of gut dysbiosis in driving disease progression through its influence on host metabolism, immune regulation, and inflammation [17], the gut microbiota has emerged as a promising therapeutic target for mitigating pollutant-induced toxicity [9,18]. Nevertheless, whether dietary supplementation with feed additives can alleviate intestinal inflammation and restore microbial homeostasis in broilers under PM_2.5_ exposure remains a question that requires further clarification.

Rosmarinic acid (RA) is a naturally occurring phenolic acid found in numerous plant species [19]. Studies have demonstrated that RA possesses antioxidant [20], anti-inflammatory [21], anti-allergic [22], anti-tumor [23], neuroprotective [24], and hepatoprotective [25] properties. Phytochemicals including RA exert intestinal protective effects by modulating the TLR/NF-κB inflammatory pathway and tight junction proteins. RA inhibits oxidative stress and inflammation by regulating the TLR4/NF-κB pathway [26,27,28] and suppresses the secretion of IL-1β, TNF-α, and IL-6 in LPS-induced acute lung injury [29]. In broilers, various plant extracts improve intestinal barrier function and alleviate inflammation in different intestinal segments: mint leaf extract restores Claudin-5 and NF-κB expression under heat stress [30]; pomegranate peel, *Sophora flavescens*, and *Artemisia annua* extracts increase Claudin-2 and reduce inflammatory cytokines [31]; a mixture of oregano, clove, and cinnamaldehyde upregulates mucin 2 and ZO-1, enhances ileal morphology, and downregulates IL-1β and IL-8 [32]; clove extract relieves *Salmonella enteritidis*-induced intestinal damage and elevates Occludin expression [33]; and astragalus polysaccharide improves intestinal morphology, upregulates tight junction proteins, inhibits pro-inflammatory cytokines, and optimizes gut microbiota [34]. Furthermore, research suggests that given this pharmacological profile, RA holds promise as a therapeutic agent for managing respiratory diseases, including allergic rhinitis [19,35,36]. Despite its well-established anti-inflammatory and barrier-protective effects, whether RA can ameliorate environmental pollutant-induced intestinal toxicity has yet to be investigated. Thus, it was hypothesized that RA would ameliorate PM_2.5_-induced intestinal damage.

To address this, a PM_2.5_-exposed broiler model was used to investigate the protective effects of RA on gastrointestinal health under PM_2.5_ exposure. This investigation focused on delineating its impacts on key endpoints: gut microbiota structure, intestinal inflammatory status, epithelial barrier function, and the TLR4/NF-κB signaling pathway. Broilers were selected as a relevant agricultural animal model susceptible to ambient air pollution to characterize intestinal responses and explore potential associations within the avian gut–lung axis.

## 2. Materials and Methods

### 2.1. Animal Ethics

This study was approved by the Animal Ethics Committee of Animal Husbandry and Veterinary Science, Henan Academy of Agricultural Sciences (Approval No. IACUC-20250915003). All procedures were performed in strict accordance with the Institute‘s Animal Care guidelines (Zhengzhou, China).

### 2.2. Animal Experiment

A total of 200 one-day-old male Arbor Acres (AA) broiler chicks were purchased and fed a corn–soybean meal-based diet formulated to meet the Aviagen Arbor Acres Broiler Nutrition Specifications (2022) during the starter phase (days 1–21) and grower phase (days 22–42) (Table 1). At 14 days of age, 144 broilers with similar body weight were randomly allocated to three groups, control (CON), PM_2.5_ exposure (PM), and PM_2.5_ exposure plus rosmarinic acid (RA), with six replicates of eight broilers each. The broilers were acclimatized in an artificial environmental control room for one week prior to the start of the experiment. The experimental phase began at 22 days of age, during which the exposure groups were transferred to three identical controlled rooms. In the PM and RA groups, PM_2.5_ (NIST 1649b) was administered at a concentration of 1000 µg/m^3^ for two hours daily via a liquid aerosol generator (NSF-6A, TOW, Shanghai, China), while the CON group was exposed to sterile saline under the same conditions. For the RA group, we supplemented the basal diet with 200 mg/kg of rosmarinic acid (10% purity, supplied by Hunan Yike Biological Technology Co., Ltd., Changsha, China). The PM_2.5_ concentration was monitored in real time throughout the exposure period (IDG100-TSP monitor, Shenzhen Weiliano Technology Co., Ltd., Shenzhen, China). The whole experiment process lasted for 21 days. Broilers were reared and managed according to the “AA Broiler Management Manual”, with free access to feed and water, and received continuous light for 24 h per day.

### 2.3. PM_2.5_ Exposure Protocol

Daily PM_2.5_ exposure was carried out from 8:00 to 10:00 (2 h per day) for the duration of the experiment. Before each exposure, broilers were moved individually into separate exposure chambers (1.2 m × 1.0 m × 0.5 m). Aerosols of physiological saline or PM_2.5_ were generated using an NSF-6A liquid aerosol system (Shanghai TOW, Shanghai, China) for whole-body inhalation. This generator uses a micro-air compressor to push high-speed airflow through the sample bottle nozzle, creating shear forces that atomize the liquid into aerosols. Larger particles are removed by inertial impaction, yielding stable aerosols with a main particle size of 2–3 μm and adjustable concentration. Airflow rate was set at 0.5 mL/min, with the nozzle directed toward the back of the birds to ensure efficient inhalation. Feed and water were covered during exposure to prevent PM_2.5_ deposition and accidental ingestion. Temperature and humidity in the exposure chambers matched the ambient conditions of the poultry house (range: 22~26 °C, 55%~65% RH), and the use of individual chambers eliminated cross-contamination between treatment groups.

### 2.4. Sample Collection and Preparation

On day 21 post-exposure, all broilers were weighed, and then from each replicate, one broiler with a weight close to the average was randomly chosen for wing vein blood collection. The blood obtained from the wing vein was centrifuged at 3000 r/min and preserved at −20 °C for further analysis. Following this, euthanasia was performed by cervical dislocation. Approximately 2 cm segments of the mid-ileum were dissected, rinsed gently with ice-cold PBS to remove intestinal contents, fixed in 4% paraformaldehyde, and stored at 4 °C for histological analysis. Cecal tissue and contents were collected and then rapidly frozen in liquid nitrogen before being stored at −80 °C for further analysis.

### 2.5. Growth Performance Measurement

Calculations of average daily gain (ADG), average daily feed intake (ADFI), and feed-to-gain ratio (F/G) were based on body weight and feed intake recorded weekly throughout the experimental period. Mortality was checked daily, and no mortality occurred during the entire trial. Therefore, no data correction was necessary.

### 2.6. Quantitative Real-Time PCR (qPCR)

RT-PCR analysis of cecal tissues was performed to assess the expression of key inflammatory markers (IL-1β, IL-6, IL-10, IFN-γ, TNF-α) and components of the TLR4 signaling pathway (TLR4, MyD88, and NF-κB p65), as well as the expression of cecal barrier junction proteins Claudin-1 (CLDN1) and Mucin 2 (MUC2) mRNA, and also Caspase3.

Table 2 shows the primer sequences designed for each gene. Around 100 mg of cecal tissue was treated with an Imagene tissue RNA rapid extraction kit (Catalog No. RE129) for the preparation of RNA samples, and the specific methods are as follows. The RNA, which was extracted, had its concentration measured by means of Nanodrop Lite (Thermo Scientific, Waltham, MA, USA). After that, the All-in-One First Strand Synthesis Master Mix (Kemix, Beijing, China) was utilized for cDNA synthesis. On a two-step real-time PCR system with a LightCycler 96 (Roche, Basel, Switzerland), 2×SYBR Green qPCR Premix (Kemix, Beijing, China) was utilized to carry out RT-qPCR. Using GAPDH as the internal reference, relative mRNA expression levels were determined by the 2^−ΔΔCt^ method.

### 2.7. 16S rRNA Sequencing and Bioinformatics Analysis of Gut Microbiota

We extracted microbial DNA from cecal samples using the E.Z.N.A.^®^ Soil DNA Kit (Omega Bio-tek, Norcross, GA, USA). DNA quality and concentration were assessed by gel electrophoresis and NanoDrop^®^ ND-2000. The V3-V4 region of the 16S rRNA gene was amplified with primers 338F and 806R on an ABI GeneAmp^®^ 9700 thermocycler (Applied Biosystems, Foster city, CA, USA). Purified amplicons from individually barcoded libraries were pooled and sequenced on the Illumina MiSeq platform following [37]. Raw data were processed on the Majorbio Cloud platform (https://cloud.majorbio.com) for diversity and correlation analyses. The raw sequences are accessible via NCBI SRA (BioProject ID PRJNA1413382).

### 2.8. Histological Examination of Ileum (H&E Staining)

Collected ileal tissues were flushed with ice-cold saline to remove luminal contents and fixed in 4% paraformaldehyde for at least 48 h. Then we processed fixed specimens through graded ethanol dehydration, xylene clearing, and paraffin embedding. Subsequently, 5 μm serial sections were cut, stained with H&E following deparaffinization and rehydration, and mounted with neutral balsam.

Morphological changes were observed and images were captured using a light microscope (BX53, Olympus, Tokyo, Japan). Five to ten randomly selected fields per section were examined at ×200 magnification.

### 2.9. Detection of TLR4, MyD88, and NF-κB Protein Expression in Cecal Tissue by ELISA

For ELISA analysis, −80 °C preserved cecal samples were homogenized in ice-cold PBS (1:9 *w*/*v*) and centrifuged (5000× *g*, 10 min). The resulting supernatants were assayed for total protein using a BCA kit, and for TLR4, MyD88, and NF-κB concentrations using commercial chicken ELISA kits, both from Nanjing Jiancheng Bioengineering Institute (Nanjing, China). All steps followed the manufacturer’s protocols.

The detailed information of the ELISA kits was as follows: Chicken Toll Like Receptor 4 (TLR4) ELISA Kit (Catalog No. H449, Lot No. 202511, Exp. 202604), Chicken Myeloid Differentiation Factor 88 (MYD88) ELISA Kit (Catalog No. H726, Lot No. 202511, Exp. 202604), and Chicken Nuclear Factor κB (NF-κB) ELISA Kit (Catalog No. H724, Lot No. 202511, Exp. 202604).

### 2.10. Statistical Analysis

The expression data of TLR4, MyD88, MUC2, NF-κB, Caspase-3, CLDN1, IL-10, IL-1β, IFN-γ, IL-6, TNF-α, ADFI, ADG, F/G, and FBW are reported as mean ± standard error (SE). For qPCR-derived gene expression, statistical comparisons were performed on ΔCt values, which were confirmed to be normally distributed and homogeneous in variance by Shapiro–Wilk and Levene’s tests, respectively. Statistical significance was determined using SPSS 26.0, with a threshold of *p* < 0.05. One-way ANOVA was conducted, and if significant overall differences among groups were detected, pairwise comparisons were further analyzed using the LSD test with Tukey’s test. Relevant graphs were generated using GraphPad Prism 5.0 software.

## 3. Results

### 3.1. RA Ameliorates PM_2.5_-Induced Impairment of Growth Performance

**Figure 1:**
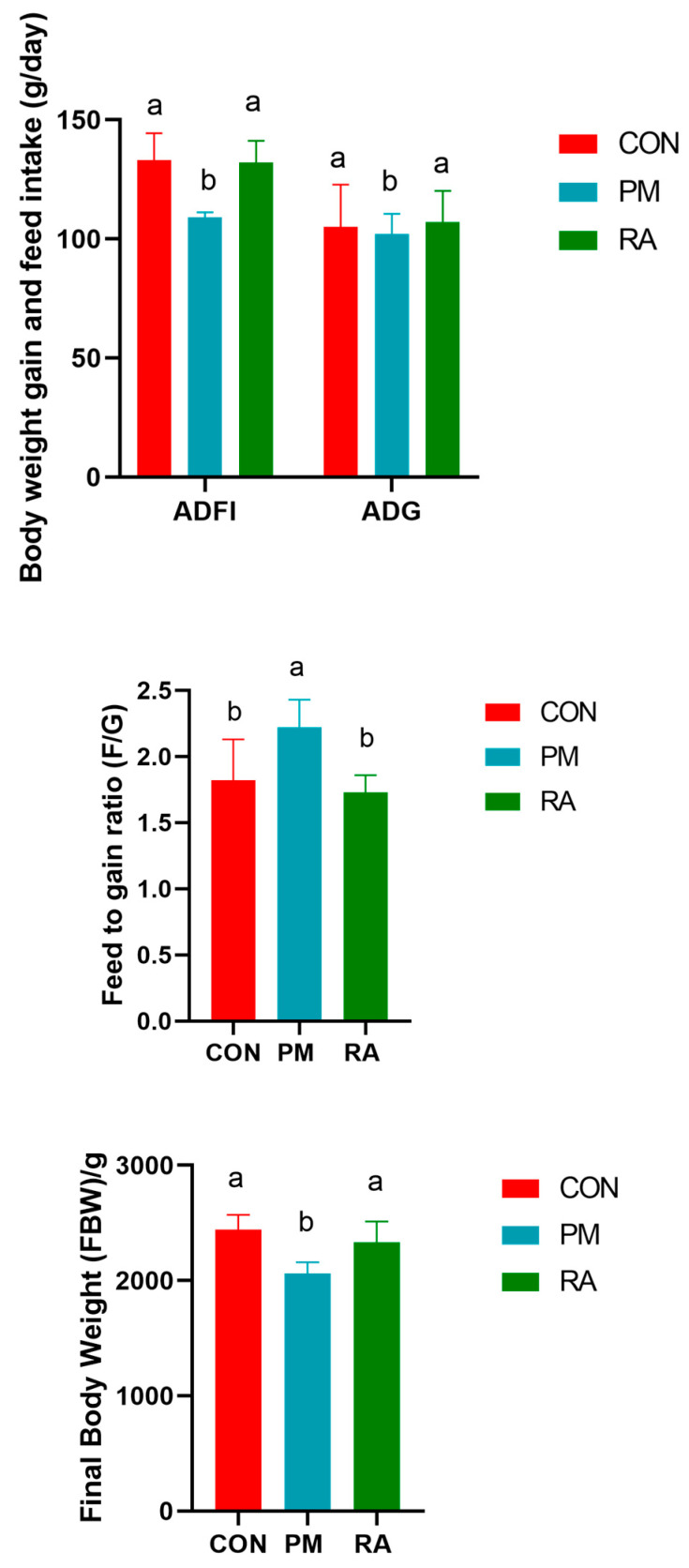
Dietary rosmarinic acid (RA) ameliorates PM_2.5_-induced impairment of growth performance. Values with different superscripts (a, b) differ significantly (*p* < 0.05).

As shown in Figure 1, dietary supplementation with RA significantly improved growth performance relative to that observed in the PM_2.5_ exposure group (*p* < 0.05). Specifically, the RA group exhibited a marked increase (*p* < 0.05) in average daily feed intake (ADFI) and average daily gain (ADG), alongside a lower (*p* < 0.05) feed-to-gain ratio (F/G). Consequently, the final body weight of RA-treated broilers at 42 days of age was also significantly greater (*p* < 0.05).

### 3.2. RA Attenuates PM_2.5_-Induced Intestinal Barrier Dysfunction

**Figure 2:**
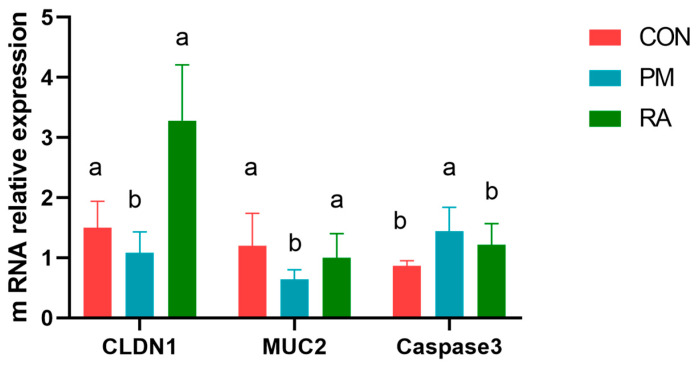
Dietary rosmarinic acid (RA) improved the gut barrier function in broilers exposed to PM_2.5_. a, b: means differ significantly (*p* < 0.05).

As shown in Figure 2, dietary supplementation with RA significantly counteracted the adverse effects of PM_2.5_ exposure on the intestinal barrier. The mRNA expression levels of the tight junction gene *CLDN1* and the mucin gene *MUC2* were upregulated (*p* < 0.05) in the RA-treated group relative to PM_2.5_ exposure. Conversely, the expression of the apoptosis-related gene caspase3 was downregulated (*p* < 0.05) by RA treatment. These results indicate that RA effectively restored the expression of key intestinal barrier components and reduced epithelial cell apoptosis induced by PM_2.5_.

### 3.3. Histopathological Changes

**Figure 3:**
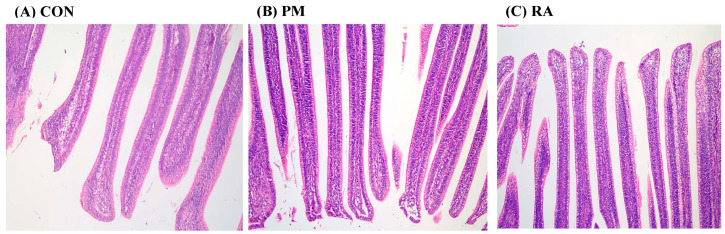
Effects of rosmarinic acid (RA) on PM_2.5_-induced histopathological changes in the broiler ileum (H&E staining; scale bar = 100 µm).

H&E staining results showed that the CON group (Figure 3A) exhibited intact and neatly arranged ileal villi, a continuous mucosal barrier, and no inflammatory infiltration or edema. In contrast, the PM_2.5_-exposed group (Figure 3B) exhibited severe pathological damage in the ileum, characterized by villus fusion and shedding, complete disruption of villus architecture, extensive inflammatory infiltration, marked edema, and severe impairment of the mucosal barrier. Compared with the PM_2.5_ group, RA treatment significantly attenuated these histopathological alterations, with only mild inflammatory infiltration, slight edema, and a largely intact mucosal barrier (Figure 3C). These observations indicate that RA effectively alleviates PM_2.5_-induced ileal tissue damage.

### 3.4. Biomarkers of Cytokine Expression

**Figure 4:**
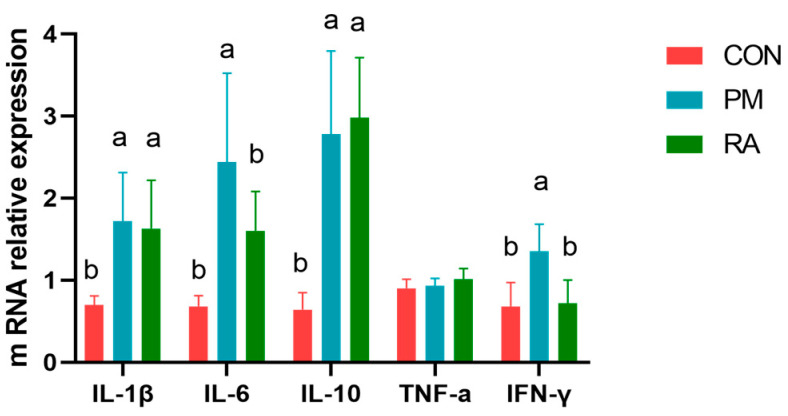
Effects of rosmarinic acid (RA) on the expression of biomarkers of cytokines in broilers exposed to PM_2.5_. a, b: means differ significantly (*p* < 0.05).

As shown in Figure 4, compared to the PM_2.5_-exposed group, RA decreased the IL-6 and IFN-γ expression (*p* < 0.05) in cecal tissues of broiler chickens under PM_2.5_ exposure. TNF-α, IL-1β, and IL-10 expression levels did not differ significantly (*p* > 0.05) between PM and RA groups.

### 3.5. TLR4 Signal Pathway

**Figure 5:**
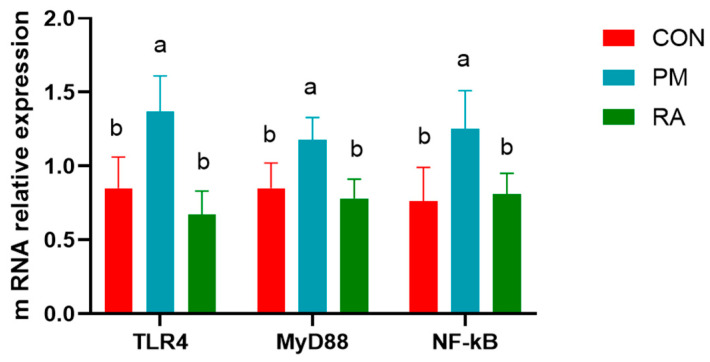
Effects of rosmarinic acid (RA) on the mRNA expression of the TLR4 signaling pathway in broilers exposed to PM_2.5_. a, b: means differ significantly (*p* < 0.05).

**Figure 6:**
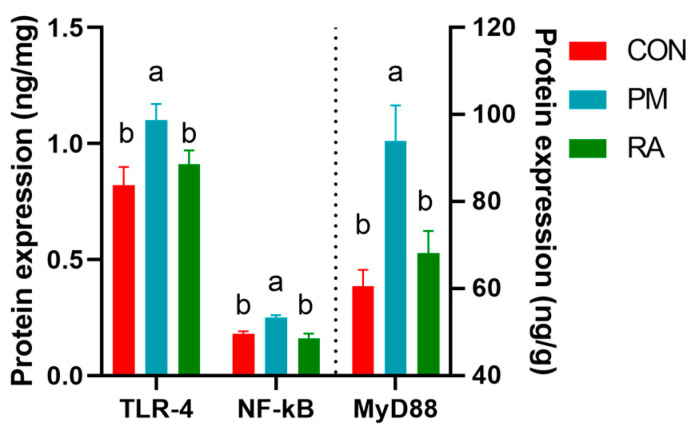
Effects of rosmarinic acid (RA) on the protein levels of the TLR4 signaling pathway in broilers exposed to PM_2.5_. a, b: means differ significantly (*p* < 0.05).

As shown in Figure 5 and Figure 6, dietary supplementation with RA significantly suppressed TLR4/NF-κB signaling following PM_2.5_ exposure. Compared to the PM_2.5_-exposed group, the RA-treated group exhibited lower (*p* < 0.05) mRNA expression and protein levels of key pathway components, including TLR4 signaling elements (MyD88, NF-κB). These data demonstrate that RA alleviates PM_2.5_-induced intestinal inflammation, at least in part, through suppression of the TLR4/MyD88/NF-κB signaling axis.

### 3.6. Alpha Diversity

**Figure 7:**
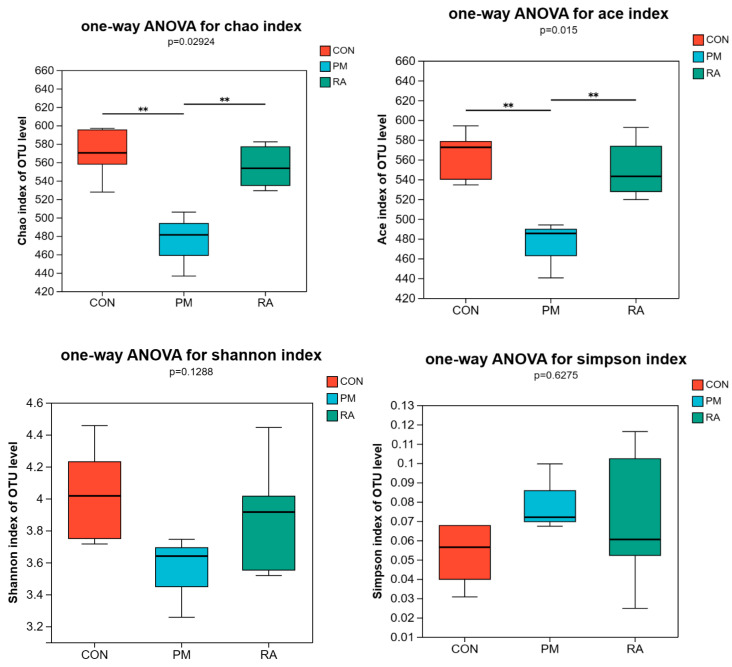
Dietary rosmarinic acid (RA) restores PM_2.5_-induced alterations in the α-diversity of gut microbiota in broilers. ** *p* < 0.01.

The indices of Chao, Shannon, Ace, and Simpson were analyzed to evaluate the α-diversity of the gut microbiota. The indicators of gut microbiota’s α-diversity in broilers are presented in Figure 7. CON and RA groups were increased (*p* < 0.05) regarding the Ace and Chao indices compared to the PM_2.5_-exposed group. Moreover, no significant difference were found among the CON, PM_2.5_-exposed, and RA groups in Shannon and Simpson indices (*p* > 0.05).

### 3.7. Beta Diversity

**Figure 8:**
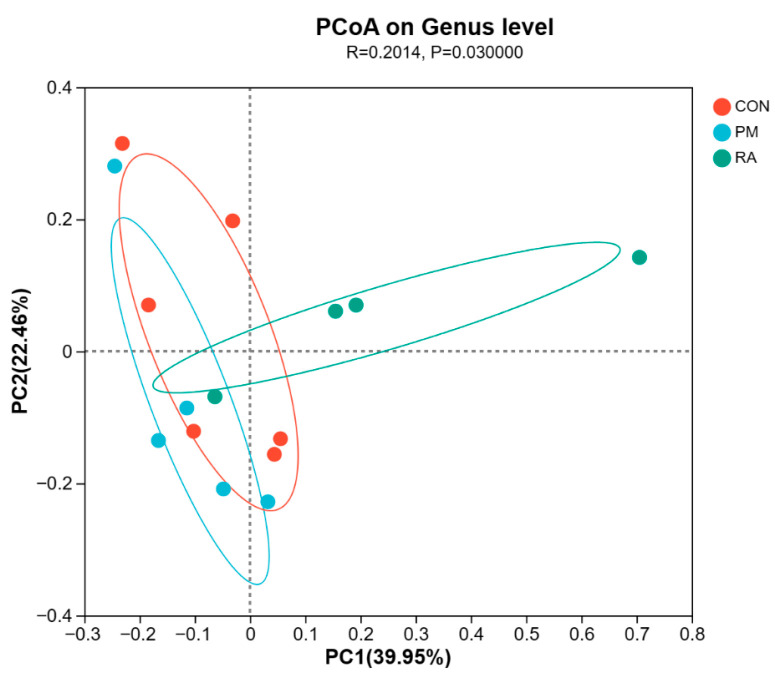
Principal Coordinate Analysis (PCoA) of gut microbial community structure (β-diversity) in broilers among the three experimental groups. The red, blue, and green ellipses are 95% confidence ellipses for the CON, PM, and RA groups, respectively, to visually show the separation and clustering of microbial community structures across the three groups.

To assess beta diversity of the gut microbiota, we performed Principal Coordinate Analysis (PCoA) based on Bray–Curtis distances for visualization (Figure 8), which was complemented by analysis of similarities (ANOSIM) for statistical validation. The ANOSIM revealed a statistically significant distinction in the overall bacterial community profiles among the three groups (R = 0.2014, *p* = 0.03).

### 3.8. Microbiota Composition

**Figure 9:**
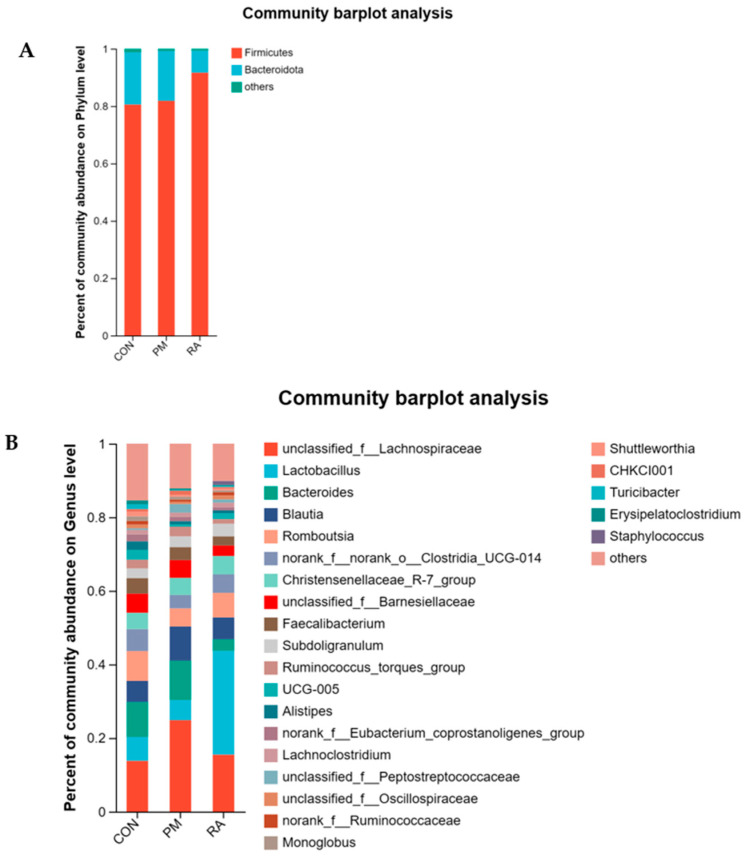
Alterations in the gut microbial composition of broilers at the phylum and genus levels in response to PM_2.5_ exposure and RA intervention. (**A**) Relative abundance of the top 10 bacterial phyla across the three experimental groups. (**B**) Relative abundance of the top 50 bacterial genera across the three experimental groups.

Then, the microbial species and their relative abundances were studied in line with the OTU from the phylum to genus levels. The dominant ones were *Firmicutes* and *Bacteroidetes* in each group on the phylum scale. RA-group levels of *Bacteroidetes* were 7.63% ± 0.31%, compared to 17.38% ± 1.68% in the PM_2.5_-exposed group, while the relative abundance of *Firmicutes* in the RA group was 91.68% ± 5.21%, compared to 81.85% ± 2.38% in the PM_2.5_-exposed group (Figure 9A).

In addition, 24 genera (*unclassified*_*f*_*Lachnospiraceae*, *Lactobacillus*, *Bacteroides*, *Blautia*, *Romboutsia*, *norank*_*f*_*norank*_*o*_*Clostridia*_*UCG*-*014*, *Christensenellaceae*_*R-7*_*group*, *unclassified*_*f*_*Barnesiellaceae*, *Faecalibacterium*, *Subdoligranulum*, *Ruminococcus*_*torques*_*group*, *UCG*-*005*, *Alistipes*, *norank*_*f*_*Eubacterium_coprostanoligenes_group*, *Lachnoclostridium*, *unclassified*_*f*_*Peptostreptococcaceae*, *unclassified*_*f*_*Oscillospiraceae*, *norank*_*f*_*Ruminococcaceae*, *Monoglobus*, *Shuttleworthia*, *CHKCI001*, *Turicibacter*, *Erysipelatoclostridium*, *Staphylococcus*) were recognized (Figure 9B). Among the 24 genera identified, certain species like those of *Bacteroides*, *Romboutsia,* and *Lactobacillus* were most prominent in the CON group, while *Blautia* had a higher proportion in the PM_2.5_-exposed group. The microbial composition of the RA group diverged from that in the group with PM_2.5_ exposure. The proportion of *Lactobacillus* in the RA group was significantly elevated relative to both CON and PM_2.5_-exposed groups. These results indicate the beneficial effect of RA on the levels of several genera, which were influenced by PM_2.5_.

### 3.9. Key Phylotypes

**Figure 10:**
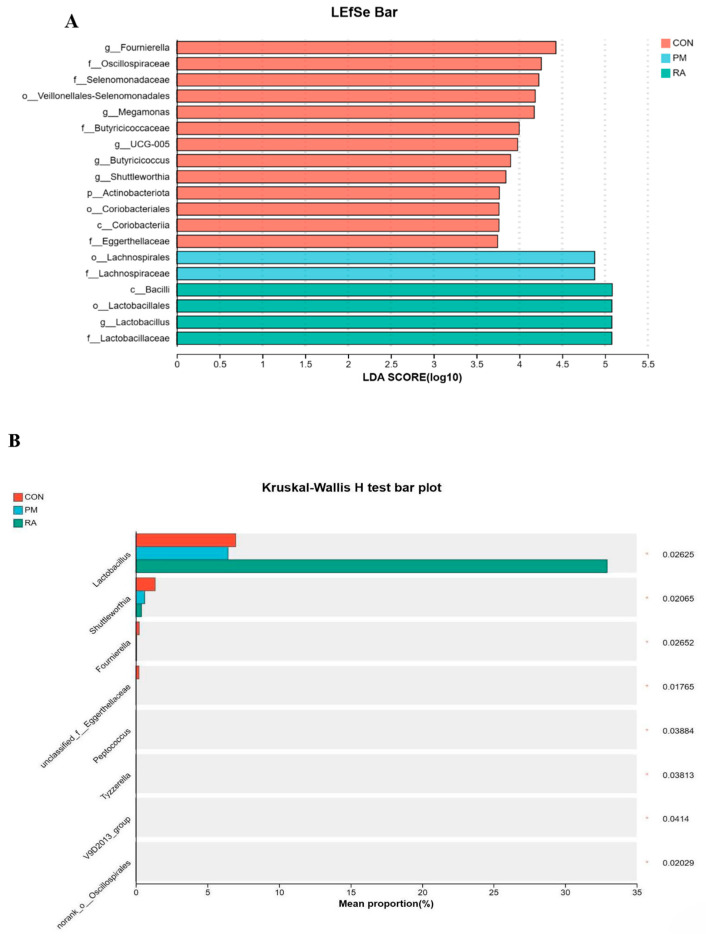
The gut microbiota in PM_2.5_-exposure broilers had their key phylotypes modulated by RA. (**A**): Linear discriminant analysis effect sizes (LEfSe) of microbial taxa are differentially abundant across three experimental groups (CON, RM, RA). Bars represent taxa with an LDA score (log_10_) > 2.0, colored by their associated group (CON: red; RM: cyan; RA: green). Taxonomic levels are indicated by prefixes (g: genus, f: family, o: order, p: phylum, c: class). (**B**): Kruskal–Wallis H test bar plot comparing the mean proportion (%) of specific microbial taxa across three experimental groups (CON: red, RM: blue, RA: green). Bars represent the mean relative abundance of each taxon, while the right-hand column displays the corresponding *p*-values from the Kruskal–Wallis test.

In order to gain deeper insight into how RA impacts the characteristic bacteria in broilers exposed to PM_2.5_, we identified bacterial taxa with differential abundance among treatments using the linear discriminant analysis effect size (LEfSe) analysis. As depicted in Figure 10A, *Fournierella*, *Oscillospiraceae*, *Selenomonadaceae*, *Veillonellales-Selenomonadales*, *Megamonas*, *Butyricicoccaceae*, *Ucg-005*, *Butyricicoccus*, *Shuttleworthia*, *Actinobacteriota*, *Coriobacteriales*, and *Eggerthellaceae* were overrepresented in the CON group, and *Lachnospirales* and *Lachnospiraceae* were overrepresented in the PM_2.5_-exposed group, while *Bacilli*, *Lactobacillales*, *Lactobacillus*, and *Lactobacillaceae* were enriched in the RA group.

In addition, to further compare the difference among the three groups, the Kruskal–Wallis H test was used to analyze the difference in bacteria. As shown in Figure 10B, there were significant differences in *Lactobacillus*, *Shuttleworthia*, *Foumierella*, *Peptococcus*, *Tyzzerella*, *V9D2013*_*group*, and *norank*_*f*_*norank*_*o*_*Oscillospirales* among the three groups.

## 4. Discussion

In this study, it was demonstrated that RA modifies the gut microbiota profile. It increases the quantity of health-promoting bacteria while reducing that of harmful bacteria. Moreover, it lessens the intestinal and systemic inflammatory responses and safeguards the gut epithelial barrier when exposed to PM_2.5_. Mechanistically, RA alleviates PM_2.5_-induced gut disorder, possibly by targeting the gut microbiota and regulating the TLR4 signaling pathway.

It has been documented in studies that RA mitigates symptoms in AR rat models exposed to PM_2.5_, an effect mechanistically related to the regulation of the NF-κB pathway and the rebalancing of Th1/Th2 [38]. Moreover, studies in a murine model of asthma have shown that RA could delay the development of airway inflammation [39]. In addition, the extensive study has demonstrated that RA could restructure the gut microbiota in mice with colitis by promoting the core microbiota and mediating the overexpression of inflammasomes [40]. The above studies indicated that RA can alleviate the inflammation in some respiratory diseases and also alleviate colitis by reshaping the microbiota; however, it is still unknown whether RA has a protective role against gut injury by optimizing the gut microbiota and reducing inflammation under PM_2.5_ exposure.

The gut is a complex ecosystem that is made up of immune cells, a mucus layer, resident microbiota, etc. Under normal circumstances, the gut’s immune system maintains tolerance and regulates appropriate immune responses to pathogens and gut microbes. Research has shown that the gut is also the target organ attacked by inhaled environmental pollutants, such as ammonia [37], leading to fluctuations in gut microbiota, inflammation, and pathological damage. It has recently been considered that gut microbiota is a key element in immune system activation, and the imbalance in the host–microbe interaction has a strong association with air pollutants. The gut microbiome may provide solutions for intestinal damage caused by air pollutants. In the current study, RA intervention significantly increased the estimated species richness (Ace and Chao indices) of the gut microbiota, whereas no marked changes were found in the Simpson and Shannon indices. These findings suggest that RA may primarily promote the colonization or recovery of certain rare species without substantially altering the overall structure of the dominant microbial communities. This provides a clue for further elucidating how RA alleviates PM_2.5_-induced damage by fine-tuning the microbiota rather than through overall restructuring.

Importantly, RA modified the structure and composition of gut microbiota under PM_2.5_ exposure. Our results showed that RA increased the *Firmicutes* abundance and decreased the *Bacteroides* abundance compared with the PM group. Research has confirmed that *Firmicutes* are related to the degradation and anti-inflammatory effects of cellulose [41], while *Bacteroidetes* belong to Gram-negative bacteria, which may become conditional pathogens under certain conditions (such as damaged intestinal barrier, imbalanced microbial community structure, decreased body resistance, etc.) [42]. At the genus level, RA significantly increased the *Lactobacillus* abundance. Lactobacillus is usually considered a potential probiotic beneficial for intestinal health, which has the function of improving the intestinal microbiota, enhancing intestinal barrier function and the body’s immune system [43]. *Oscillospira* is considered a potential SCFA-producing genus, capable of generating butyrate and other short-chain fatty acids, making it a key candidate biomarker for the selection of next-generation probiotics [16]. *V9D2013*_*group* was also found to be capable of butyrate production [44,45]. Studies have shown that intestinal infections induced by *Eimeria tenella* [46] or *Salmonella typhimurium* [47] in broilers increase the abundance of *Shuttleworthia*, indicating that *Shuttleworthia* may be a harmful bacterium [48]. Our results suggest that the RA increased the relative abundance of potential probiotics such as *Lactobacilli*, *V9D2013*_*group*, and *Oscillospirale* abundance in cecal contents, reduced the relative abundance of opportunistic pathogenic bacteria in *Shuttleworthia,* and optimized the structure of the cecal microbiota.

It has been demonstrated by a great number of studies that the colonization of specific probiotics like *Lactobacillus* and *Bifidobacterium* is able to cause an increase in the concentration of SCFAs. This is helpful for relieving intestinal inflammatory reactions, enhancing the mucosal barrier function, and withstanding the invasion of pathogens [49,50]. The gut microbiota can form a microbial barrier, which is among the four main barriers for gut barrier function and is crucial for intestinal health. In this research, the quantity of lactic acid bacteria in the RA group showed a significant rise, which means that RA is able to relieve the intestinal mucosal damage induced by PM_2.5_, decrease intestinal permeability, and strengthen intestinal barrier function. The epithelial barrier can be regulated by Claudin1. It safeguards the intestinal barrier function and stops pathogens and harmful substances from migrating and invading the cell membrane in the intestine [51].

The mucosal barrier consists of an outer mucus layer, primarily formed by goblet cell-derived MUC2 mucin, and an inner single-layer epithelium [52]. Barrier function is tightly controlled by tight junctions (TJs), the key intercellular structures regulating paracellular permeability [53]. Under stress conditions—such as heat stress or pathogenic infection—impairment of these components compromises barrier integrity, which can reduce growth performance and increase disease susceptibility in broilers, as underscored by foundational research on poultry gut immunity and barrier health [54]. Claudin-1 is widely recognized as a critical biomarker of intestinal barrier integrity in broilers facing both pathogenic and environmental stressors [55,56]. Similarly, MUC2 not only constitutes a structural barrier but also plays a central role in mediating host–microbe interactions in stressed poultry [57,58]. Supporting its functional importance, MUC2-deficient mice exhibit elevated pro-inflammatory cytokine production and are prone to spontaneous colitis [59]. Numerous studies have demonstrated that phytobiotics effectively improve intestinal barrier function in broilers. At the molecular level, plant extracts such as mint leaf have been shown to restore Claudin-5 and NF-κB expression in heat-stressed broilers [30], while resveratrol, oregano, clove, and cinnamaldehyde can upregulate the expression of critical tight junction proteins including CLDN1, Occludin (OCLN), ZO-1, and mucin 2 [32,60]. At the tissue level, these bioactive compounds also enhance intestinal villus height and the villus height-to-crypt depth ratio, thereby strengthening the intestinal mucosal barrier [34]. Furthermore, plant extracts can increase the abundance and diversity of the intestinal microbiota, stabilize gut homeostasis, and further preserve barrier integrity and intestinal morphology [61]. In the present study, RA significantly increased the expression of CLDN1 and MUC2 genes compared to the PM_2.5_-exposed group. This finding suggests that RA enhances intestinal barrier function in broilers under particulate matter stress. Although short-chain fatty acid (SCFA) concentrations were not directly quantified here, the observed increase in SCFA-associated microbiota abundance—*Lactobacillus*, *Oscillospiraceae*, and *V9D2013*_*group*—offers a plausible microbial explanation for the improved barrier function and attenuated inflammatory response. We hypothesize that RA promotes a gut microbiota profile conducive to SCFA synthesis, particularly butyrate, which is known to support epithelial integrity [62,63] and mitigate inflammation [64,65]. Thus, enriching SCFA-producing bacteria may represent one of the indirect mechanisms through which RA alleviates intestinal injury.

The level of intestinal inflammatory factors reflects the local immune status, where the pro-/anti-inflammatory cytokine balance maintains gut homeostasis. The inflammatory response is largely governed by the signaling cascade initiated and propagated by key pro-inflammatory cytokines, notably IL-1β, IL-6, and TNF-α [66,67,68]. In poultry, these cytokines are similarly involved in intestinal immune responses and are associated with disease states such as necrotic enteritis and coccidial infection [69,70]. Critical for immune homeostasis, cytokines such as IL-10 play a vital role in dampening inflammatory responses and orchestrating the process of tissue regeneration [71,72], an effect that has also been validated in avian inflammatory models [73]. Multiple phytobiotics can alleviate intestinal inflammation by regulating gene expression in the TLR/NF-κB pathway. Quercetin inhibits excessive activation of the TLR-MyD88-NF-κB pathway, upregulates AvBD expression, and modulates the gut microbiota to lower mortality in broilers [74]. Protocatechuic acid blocks abnormal activation of the TLR4/p38 MAPK and NF-κB pathways, thereby relieving oxidative stress and inflammatory imbalance caused by heat stress [75]. Dietary IA alleviates LPS-induced intestinal injury by balancing the TLR4/MyD88/NF-κB inflammatory pathway and the Nrf2 antioxidant pathway [76]. MCE reduces overexpression of jejunal TLR2 and the release of IFN-γ and IL-17, repairs the intestinal barrier, and restores microecological homeostasis [77]. Isoquinoline alkaloids inhibit the TLR-MyD88-NF-κB signaling axis, mitigate cecal inflammation, and upregulate antioxidant and barrier-related genes in the duodenum [78]. Our results suggest that dietary supplementation with RA significantly downregulated IL-6 and IFN-γ levels in the intestines of PM_2.5_-exposed broilers, indicating that RA may alleviate cecal inflammation by inhibiting the secretion of key inflammatory mediators. This effect may be related to the pharmacological activity of RA and its modulation of the gut microbiota. In poultry research, certain plant extracts have been reported to improve intestinal health by shifting the balance from a pro-inflammatory to an anti-inflammatory milieu by dampening key cytokines [79,80]. Mechanistically, RA mediates its anti-inflammatory effects through the suppression of the TLR4/NF-κB signaling cascade. In the present study, intestinal TLR4, MyD88, and NF-κB levels were markedly reduced by RA in broilers following PM_2.5_ exposure. This aligns with observations in poultry, where other natural products confer protection by alleviating intestinal inflammation and enhancing barrier integrity by suppressing the TLR4/NF-κB axis [81,82,83]. Taken together, these results suggest that RA may mitigate PM_2.5_-induced intestinal inflammation in broilers by suppressing the TLR4/NF-κB pathway and modulating the gut microbiota.

In addition, the present study also reveals that RA significantly alleviates the decline in growth performance of broilers induced by PM_2.5_ exposure. Dietary supplementation with RA notably increased the ADFI and ADG of broilers, while significantly reducing the F/G, ultimately leading to a significant improvement in body weight at 42 days of age. These results indicate that RA not only effectively counteracts the growth suppression potentially caused by PM_2.5_ but also actively improves feed conversion efficiency. We hypothesize that this improvement in growth performance is not an isolated phenomenon but is likely closely related to the optimized intestinal microbiota structure and alleviated systemic inflammation observed in this study. Specifically, the enrichment of beneficial bacteria such as Lactobacillus in the intestine following RA intervention is typically associated with enhanced intestinal barrier function, increased SCFA production, and improved nutrient digestion and absorption efficiency. Concurrently, the downregulation of key inflammatory pathway factors related to TLR4/NF-κB observed in the RA group indicates a mitigation of systemic low-grade inflammation. Under PM_2.5_-induced stress conditions, animals often need to allocate more energy toward immune responses, thereby diverting resources away from growth. Therefore, RA may effectively reduce the “immune–metabolic cost” triggered by environmental stress through the “microbiota–immune–metabolism” axis, directing more energy and nutrients toward protein deposition and muscle growth. This directly explains the synergistic improvement of increased ADG and decreased F/G. This approach stabilizes and enhances broiler performance in suboptimal health environments by modulating host intestinal health and immune balance, rather than relying on exogenous antibiotics or growth promoters, aligning with the current trend in animal husbandry toward green and healthy farming practices.

While this study provides valuable insights into the protection of RA in gut health under PM_2.5_ exposure, it is not without limitations. First, the sample sizes for slaughter performance and key assays (*n* = 6 per group) were limited by housing and welfare considerations, which may affect the detection of subtle effects. Second, the experimental design lacked a standalone RA control group, which prevents us from distinguishing the baseline effects of RA alone from its interactive effects under PM2.5 exposure; this limits the mechanistic interpretation of RA as a protective agent, particularly given its well-documented antioxidant properties. Third, the present study did not directly measure PM_2.5_ in cecal contents or feces, making it impossible to definitively distinguish between systemic effects originating from pulmonary exposure and local effects caused by direct ingestion and accumulation of PM_2.5_ in the gastrointestinal tract—particularly relevant given that ingestion is a physiologically plausible exposure route in poultry. Furthermore, the present study demonstrates strong associations between RA supplementation, microbial shifts, immune modulation, and improved growth, but cannot establish definitive causality. Future work employing larger cohorts and direct mechanistic approaches—such as fecal microbiota transplantation, microbiota depletion models, or TLR4-specific inhibitors—is needed to elucidate causal pathways within the gut–immune–growth axis and to validate these findings under varied real-world conditions.

## 5. Conclusions

In conclusion, the present study demonstrates that dietary rosmarinic acid (RA) plays a pivotal protective role in alleviating PM_2.5_-induced intestinal impairment in broilers. Specifically, RA effectively maintains intestinal barrier integrity under PM_2.5_ exposure, which is directly supported by the upregulated mRNA expression of the tight junction protein gene CLDN1 and the mucin gene MUC2—key mediators of intestinal epithelial barrier function. Furthermore, RA exerts a potent anti-inflammatory effect by suppressing the TLR4 signaling pathway, as evidenced by the downregulated expression of TLR4 pathway-related genes and pro-inflammatory cytokines (IL-6 and IFN-γ). At the gut microbial level, RA also functions as a critical modulator: it enhances the relative abundance of beneficial probiotics (e.g., *Lactobacillus*, *V9D2013_group*, and *Oscillospirale*) while reducing the colonization of opportunistic pathogens such as *Shuttleworthia* in the cecum. Collectively, these findings confirm that RA mitigates PM_2.5_-induced intestinal injury and microbiota dysbiosis in broilers through synergistically regulating intestinal barrier function, inflammatory response, and gut microbial homeostasis.

## Figures and Tables

**Table 1:** Ingredients and nutrients of the basal diet (%, as-fed basis).

Items	1 to 3 Weeks	4 to 6 Weeks
Ingredients		
Corn	58.61	61.31
Soybean meal	33.48	30.49
Soybean oil	3.60	4.49
Limestone	1.32	1.34
CaHPO4	1.75	1.40
NaCl	0.29	0.30
Lys	0.24	0.14
Met	0.27	0.13
Choline chloride	0.20	0.17
Premix ^(1)^	0.24	0.23
Total	100.00	100.00
Nutrient levels		
ME/(MJ/kg) ^(2)^	12.35	12.89
CP	20.76	19.44
Ca	1.08	0.98
AP	0.64	0.58
Met	0.47	0.44

^(1)^ The premix was given for 1–3 wk provided the following nutrients per kilogram of diet: Vitamin A, 12,500 IU; Vitamin D_3_, 3750 IU; Vitamin E, 16 IU; Vitamin K_3_, 2.0 mg; Vitamin B_1_, 2.5 mg; Vitamin B_2_, 8 mg; Vitamin B_6_, 2.5 mg; Vitamin B_12_, 0.015 mg, Panthoenic acid calcium, 12.5 mg; Nicotinic acid, 32.5 mg; Folic acid, 1.25 mg; Biotin, 0.125 mg; Choline, 700 mg; Zn (ZnSO_4_·7H_2_O), 60 mg; Fe (FeSO_4_·7H_2_O), 80 mg; Cu (CuSO_4_·5H_2_O), 8 mg; Mn (MnSO_4_·H_2_O), 110 mg; I (KI), 0.35 mg; Se (Na_2_SeO_3_), 0.15 mg. The premix was given for 4–6 wk provided the following nutrients per kilogram of diet: Vitamin A, 10,000 IU; Vitamin D_3_, 3400 IU; Vitamin E, 16 IU; Vitamin K_3_, 2.0 mg; Vitamin B_1_, 2.0 mg; Vitamin B_2_, 6.4 mg; Vitamin B_6_, 2.0 mg; Vitamin B_12_, 0.012 mg; pantothenic acid calcium, 10 mg; nicotinic acid, 26 mg; folic acid, 1 mg; biotin, 0.1 mg; choline, 500 mg; Zn (ZnSO_4_·7H_2_O), 40 mg; Fe (FeSO_4_·7H_2_O), 80 mg; Cu (CuSO_4_·5H_2_O), 8 mg; Mn (MnSO_4_·H_2_O), 80 mg; I (KI), 0.35 mg; Se (Na_2_SeO_3_), 0.15 mg. ^(2)^ metabolizable energy was calculated, whereas the others were measured.

**Table 2:** The primer sequence of the target gene.

Target Gene	Primer Sequence (5′ to 3′)	Length	Login ID
GAPDH	F:TGAAAGTCGGAGTCAACGGAT	230 bp	NM_204305.1
	R:ACGCTCCTGGAAGATAGTGAT		
TLR4	F:ACGGAAGGCTTTGGTTGGGATT	184 bp	NM_001030693.1
	R:GATGTTGCTATCTGGTGCTTGGAA		
MyD88	F: TCTGGTGACTGTGGAGCAAGGAA	206 bp	NM_001030962.4
	R: CCGCTTGTAGGAAGGCACTAATGG		
NF-κB	F: TCATCCACCGCCGCCACATT	232 bp	NM_205129.1
	R: GGCTGAGGAAGGCACTGAAGTC		
IL-1β	F:AGAAGAAGCCTCGCCTGGAT	131 bp	NM_204524.1
	R:CCTCCGCAGCAGTTTGGT		
IFN-γ	F:AGTCAAAGCCGCACATCAAACAC	133 bp	NM_205149.1
	R:CGCTGGATTCTCAAGTCGTTCATC		
TNF-α	F:GGACAGCCTATGCCAACAAG	168 bp	NM_204267.1
	R:ACACGACAGCCAAGTCAACG		
IL-6	F:CCTCCTCGCCAATCTGAAGTCA	210 bp	NM_204628.1
	R:AACGGAACAACACTGCCATCTG		
IL-10	F:ATCCAACTGCTCAGCTCTGAACTG	101 bp	NM_001004414.2
	R:GGCAGGACCTCATCTGTGTAGAAG		
*CLDN1*	F:GACCAGGTGAAGAAGATGCGGATG	107 bp	NM_001013611.2
	R:CGAGCCACTCTGTTGCCATACC		
*MUC2*	F:AGGTAATTGTCTGGCCGTGG	111 bp	NM_001318434.1
	R:GTGGTTGTACCTTCGGTGCT		
Caspase3	F:CGCAGAGTGGCAGAATATGAATCC	220 bp	NM_204725.1
	R:ATGGCAGTGAACAGCAAGTCAGA		

## Data Availability

The original contributions presented in this study are included in the article material. Further inquiries can be directed to the corresponding author.
